# Quantifying drivers of wild pig movement across multiple spatial and temporal scales

**DOI:** 10.1186/s40462-017-0105-1

**Published:** 2017-06-15

**Authors:** Shannon L. Kay, Justin W. Fischer, Andrew J. Monaghan, James C. Beasley, Raoul Boughton, Tyler A. Campbell, Susan M. Cooper, Stephen S. Ditchkoff, Steve B. Hartley, John C. Kilgo, Samantha M. Wisely, A. Christy Wyckoff, Kurt C. VerCauteren, Kim M. Pepin

**Affiliations:** 1 United States Department of Agriculture, Animal Plant Health Inspection Service, Wildlife Services, National Wildlife Research Center, 4101 LaPorte Avenue, Fort Collins, CO 80521-2154 USA; 2 Research Applications Laboratory, National Center for Atmospheric Research, Boulder, CO 80305 USA; 3 Savannah River Ecology Laboratory, Aiken, SC 29802 USA; 4 Warnell School of Forestry and Natural Resources, Athens, GA 30602 USA; 5 Range Cattle Research and Education Center, 3401 Experiment Station, Ona, FL 33865 USA; 6 East Foundation, 200 Concord Plaza Drive, Suite 410, San Antonio, TX 78216 USA; 7 Texas AgriLife Research, Texas A&M University System, 1619 Garner Field Road, Uvalde, TX 78801 USA; 8 School of Forestry and Wildlife Sciences, Auburn University, 3301 Forestry and Wildlife Sciences Building, Auburn, AL 36849 USA; 9 United States Geological Survey, Wetland and Aquatic Research Center, 700 Cajundome Blvd, Lafayette, LA 70506 USA; 10 United State Department of Agriculture, Forest Service, Southern Research Station, P.O. Box 700, New Ellenton, SC 29809 USA; 11 Department of Wildlife Ecology and Conservation, University of Florida, Gainesville, FL 32611-0430 USA; 12 Caesar Kleberg Wildlife Research Institute, Texas A&M University–Kingsville, Kingsville, TX 78363 USA; 13 Santa Lucia Conservancy, 26700 Rancho San Carlos Rd, Carmel, CA 93923 USA

**Keywords:** Animal movement, Reaction norm, Feral swine, GPS, Home range, Wild pig, *Sus scrofa*, MCP, AKDE

## Abstract

**Background:**

The movement behavior of an animal is determined by extrinsic and intrinsic factors that operate at multiple spatio-temporal scales, yet much of our knowledge of animal movement comes from studies that examine only one or two scales concurrently. Understanding the drivers of animal movement across multiple scales is crucial for understanding the fundamentals of movement ecology, predicting changes in distribution, describing disease dynamics, and identifying efficient methods of wildlife conservation and management.

**Methods:**

We obtained over 400,000 GPS locations of wild pigs from 13 different studies spanning six states in southern U.S.A., and quantified movement rates and home range size within a single analytical framework. We used a generalized additive mixed model framework to quantify the effects of five broad predictor categories on movement: individual-level attributes, geographic factors, landscape attributes, meteorological conditions, and temporal variables. We examined effects of predictors across three temporal scales: daily, monthly, and using all data during the study period. We considered both local environmental factors such as daily weather data and distance to various resources on the landscape, as well as factors acting at a broader spatial scale such as ecoregion and season.

**Results:**

We found meteorological variables (temperature and pressure), landscape features (distance to water sources), a broad-scale geographic factor (ecoregion), and individual-level characteristics (sex-age class), drove wild pig movement across all scales, but both the magnitude and shape of covariate relationships to movement differed across temporal scales.

**Conclusions:**

The analytical framework we present can be used to assess movement patterns arising from multiple data sources for a range of species while accounting for spatio-temporal correlations. Our analyses show the magnitude by which reaction norms can change based on the temporal scale of response data, illustrating the importance of appropriately defining temporal scales of both the movement response and covariates depending on the intended implications of research (e.g., predicting effects of movement due to climate change versus planning local-scale management). We argue that consideration of multiple spatial scales within the same framework (rather than comparing across separate studies *post-hoc*) gives a more accurate quantification of cross-scale spatial effects by appropriately accounting for error correlation.

**Electronic supplementary material:**

The online version of this article (doi:10.1186/s40462-017-0105-1) contains supplementary material, which is available to authorized users.

## Background

Movement capacity, or an individual’s tendency to move based on various internal and external factors, is a central component of animal movement ecology [1]. Understanding the drivers of this capacity can assist in guiding decisions about how to best conserve or manage animal populations [2–4], in predicting how climate change and anthropogenic influences will affect wildlife population dynamics [5, 6] and in predicting disease spread [7]. Recent technological advances in GPS devices have allowed for the collection of increasingly fine-scaled location data, and have resulted in a rich source of information for quantifying animal movement capacity. However, as costs increase dramatically with GPS fix resolution, the duration of the study, and the number of individuals collared, it is often not feasible to have a large sample size of long-term, high-resolution data. Thus, much of our knowledge of animal movement capacity comes from studies which focus on relatively small sample sizes and a single ecological scale, despite the fact that the capacity for animals to move is determined by the interaction of many factors acting on numerous spatial and temporal scales [8, 9]. By examining movement capacity across multiple spatio-temporal scales within a single analytical framework, we can identify general trends in animal movement, quantify factors that limit movement, and gain a more in-depth understanding of how multi-scale factors interact in driving movement capacity.

Ecologists often deal with a variety of processes that act on numerous temporal and spatial scales. Identifying the appropriate scale (s) within which to test hypotheses about ecological mechanisms continues to be a developing ideology in ecology [10–12]. In many cases, one scale of data is analyzed and used to extrapolate to other scales. For example, “top-down” or “bottom-up” approaches where population-level analyses are used to extrapolate to the individual-level, or individual-level analyses are used to extrapolate to the population-level [13]. However, environmental processes can be more influential at one scale than another. For instance, [14] found movement responses of woodland caribou were scale-dependent when they classified movement as either interpatch or intrapatch, such as cover type which had a greater effect on intrapatch movements than interpatch movements. Furthermore, movement responses may differ based on geographic location, and hence, environmental factors may have stronger effects depending on evolutionary adaptations to local resources [15]. Therefore, examining factors that affect animal movement across different temporal and spatial scales provides a more thorough understanding of movement capacity in the broader context of animal movement behavior.

To gain a better understanding of how multiple spatio-temporal scales affect animal movement, we examined movement capacity of wild pigs (*Sus scrofa*) using GPS telemetry data from 14 studies [16–20] conducted across the southern U.S. from 2004 to 2014. Wild pigs are an invasive species that cause a substantial amount of crop and property damage [16–20] and pose a significant disease threat to livestock operations [21–24], which could have cataclysmic effects on the economy. Wild pig populations are abundant across much of the U.S. in a variety of habitats, and appear to have few constraints on habitat invasion, making them an ideal study species for examining movement capacity across ecological scales. It’s suggested that resource abundance and distribution affect the movement behavior of wild pigs, where they can reduce their energy-expenditure and travel less when resources are readily available and plentiful [25]. Additionally, it has also been suggested that anthropogenic alterations to the landscape such as development [26] and using bait for trapping [27] may modify wild pig movement patterns. Thus, by pooling data from various regions and examining movement across a large geographical gradient, we are able to identify factors that affect wild pig movement capacity across landscapes, and predict how they may constrain movement locally. Prediction of movement levels can be useful in planning management actions (i.e., optimal time and places to find individuals) and preventing invasions (i.e., understanding how far individuals may move during new invasions in different habitats). Furthermore, wild pigs are one of the least studied ungulate species in terms of their movement ecology, with many wild pig movement studies examining genetic differences between populations [28]. Of the few studies that focused on wild pig movement ecology (e.g., [29–33]), most were generally limited in spatial or temporal scope (although see [34]). Thus, our basic understanding of factors affecting wild pig movement capacity across their distribution is weak, making it challenging to predict movement constraints.

To address gaps in our fundamental understanding of movement capacity across spatio-temporal scales, we examined movement capacity of wild pigs at three different temporal scales: daily, monthly, and overall. We measured movement capacity using three simple metrics, including the maximum distance moved in a day (MxD), the average distance moved in an hour (MHD), and home range size. The MxD response represents the maximum straight-line distance we would expect an individual to travel in a day during typical daily activities (e.g., foraging and mating). The MHD response is commonly used in wildlife movement analyses to reflect short-term movement habits, and average rate of speed [31, 35, 36]. We also used home range size as an additional response to represent the size of the area in which wild pigs tend to exploit at both the monthly and overall scales. Collectively, these movement metrics can help managers design optimal control strategies by quantifying the effects of factors driving wild pig movement, and predicting movement trends in areas that have been recently invaded.

Based on previous work [25, 34], we hypothesized that five types of predictor variables may predict the movement ecology of wild pigs: meteorological, landscape, geographic, individual-based and temporal. We also hypothesized that the importance of different environmental predictors would differ across temporal scales of movement. For example, we hypothesized that movement capacity on the daily scale would be affected by current environmental conditions that could reflect weather events (e.g., temperature, precipitation, barometric pressure, wind speed, saturation, humidity) because these factors have been shown to affect movement in other wildlife species [31, 37–42]. In contrast, we hypothesized that movement at the monthly and overall scales would be influenced by biotic productivity (i.e., measured by weather patterns over longer timescales), landscape features such as distances to various types of resources, and time of the year, because these factors likely affect the seasonal variation in movement due to resource availability [43, 44]. Studies have shown differences in social behavior between male and female wild pigs [45, 46], so we hypothesized that individual-level characteristics such as sex and age would be important at all scales. In addition, we hypothesized that broad-level geographic characteristics (i.e., ecoregion) would also explain variation in movement patterns because these factors encompass numerous variables that act on many different scales. Thus, we expected ecoregion could be useful for predicting movement levels and shaping management practices regionally.

We employed a two-step approach to analyzing movement rates and home range sizes by first using a machine-learning algorithm, random forest regression, to identify the most important factors affecting our response variables on each scale. We then fit generalized additive mixed models (GAMMs) using the resulting predictor variables from the machine-learning algorithm to quantify effects of covariates while accounting for spatio-temporal and biological correlations. Our results describe drivers of movement capacity that can aid managers in identifying the most effective techniques and spatio-temporal implementation strategies for successful population control. Furthermore, our analytical approach should inform future large-scale movement studies using diverse datasets, and our results are relevant to predicting the effects of future urban development and climate change on animal movement.

## Methods

### Data processing

We analyzed GPS data from individual pigs in studies across six states including Florida, Georgia, Louisiana, Missouri, South Carolina, and Texas. The full dataset included more than 400,000 total locations (Additional file 1: Figures S1 and S2) arising from 226 individuals that were monitored from 2004 to 2016. Individuals consisted of both male and female sub-adult and adult wild pigs, as well as two juvenile (i.e., < 1 year old) females. Note that 89/226 individuals did not have an associated age class. Monitoring times varied considerably across studies, with some individuals only monitored for a few days and others monitored for almost 2 years. However, most individuals were monitored for 2–4 months. Temporal resolution between GPS fixes varied substantially across studies (from locations recorded every 15 min to an average of two locations per day), although there were no strong trends between movement metrics and fix rates (Additional file 1: Figure S3). The average number of locations per pig per day was 14, which is approximately one relocation every 1.5 h. We accounted for the variable fix rates through various combinations of random effects and weighting structures, which are described in detail in the following section.

Data were first screened by requiring that each individual was (1) monitored for at least 20 days, (2) had at least two recorded locations per day, and (3) that each monthly average movement rate or home range size estimate was based on at least 15 days of monitoring. Therefore, days which had fewer than two locations, and months for which the individual was not monitored for at least 15 days were discarded. Locations with dilution of precision (DOP) values greater than 10 were also removed to ensure at least moderate accuracy of locations [47]. Finally, since it is generally accepted that wild pig activity is primarily nocturnal due to hunting and heat avoidance [46, 48], we only used nightly locations in our analyses which were defined to be between 6 pm and 8 am the following morning. Thus, all references to “daily movement” are in fact reflective of nocturnal and crepuscular movement.

### Response variables

**Fig. 1:**
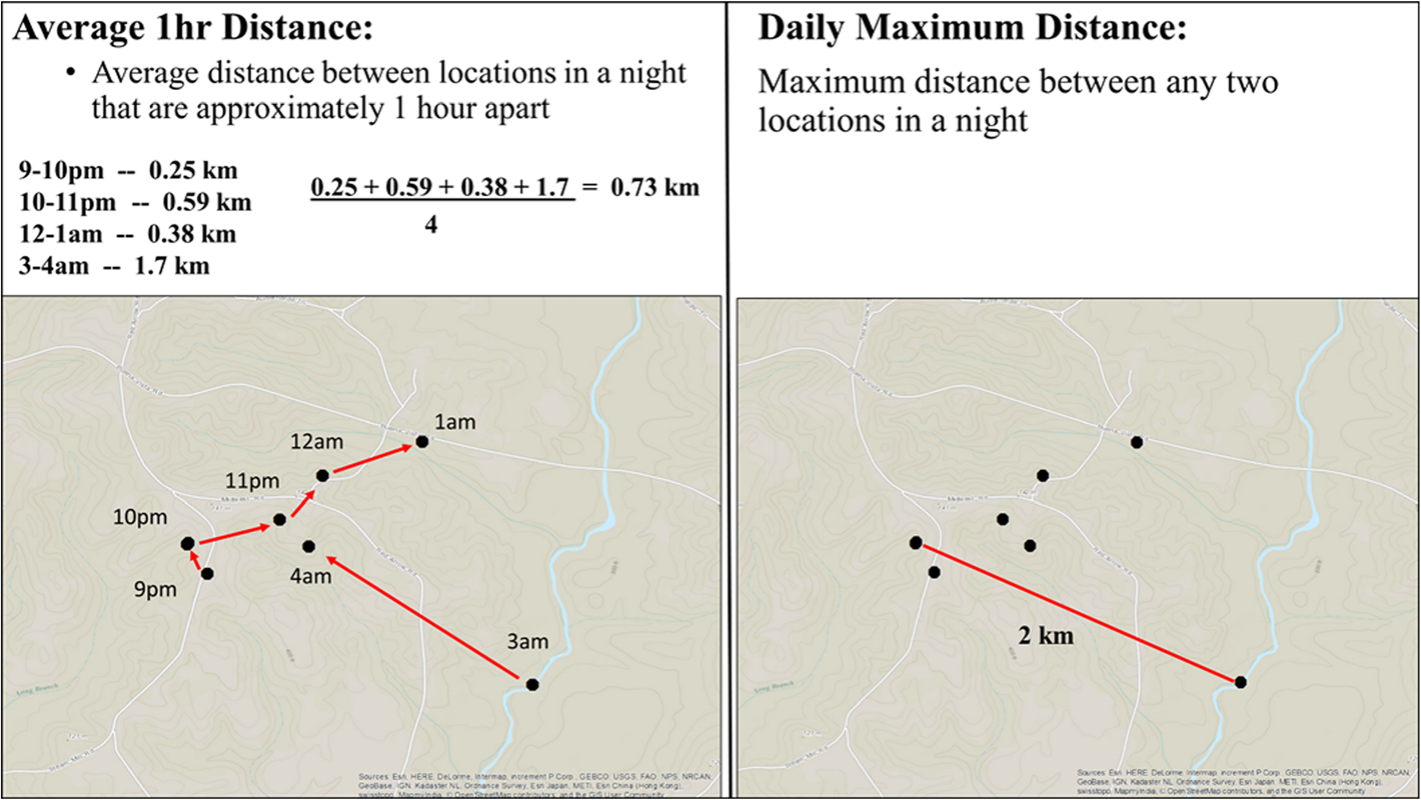
Schematic illustrating how daily movement values of wild pigs were calculated. Maximum distance is defined as the maximum distance between any two locations in a day (*right panel*) while average 1 h distance is calculated by averaging distances that are approximately 1 h apart (*left panel*)

Two types of movement values were assessed at all three temporal scales (daily, monthly, and overall): MxD and MHD (Fig. 1). MxD was defined to be the maximum distance between any two locations in a day, and represented a general movement metric that reflected the farthest distance an individual would travel in one night (e.g., searching for food or mates). Most of these daily maximum distances (approximately 95%) were based on telemetry fixes that were more than 3 h apart, and did not appear to have any relationship with fix rate (Additional file 1: Figure S3). MHD was calculated by averaging distances that were approximately 1 h apart within a day (Fig. 1), and provided an idea of how much an individual moved in a short time period (e.g., foraging). For an arbitrary example, if an individual had fixes every half hour starting at 8:00 and ending at 10:30, four distances would be used to calculate the average MHD (two from on the hours at 8, 9, and two more from the half hour at 8:30 and 9:30). Therefore, sample sizes differed between the two movement responses because MxD only required two locations per day (*n* = 227 individuals) whereas the MHD required at least two sequential locations within approximately 1 h of each other per day (*n* = 180 individuals from all studies except one from Texas). Consequently, the mean fix rate per pig per day for MHD was 17, which was slightly higher than average fix rate of 14 for all pigs. However, the median fix rates of 12 locations/pig/day and 13 locations/pig/day were almost identical between the two groups. We then averaged daily movement values for each individual at the monthly and overall (all values combined) scales. Together, these movement metrics provide an indication of how much and how far an individual tends to travel in a day, which helps us understand an individual’s daily movement capacity.

**Fig. 2:**
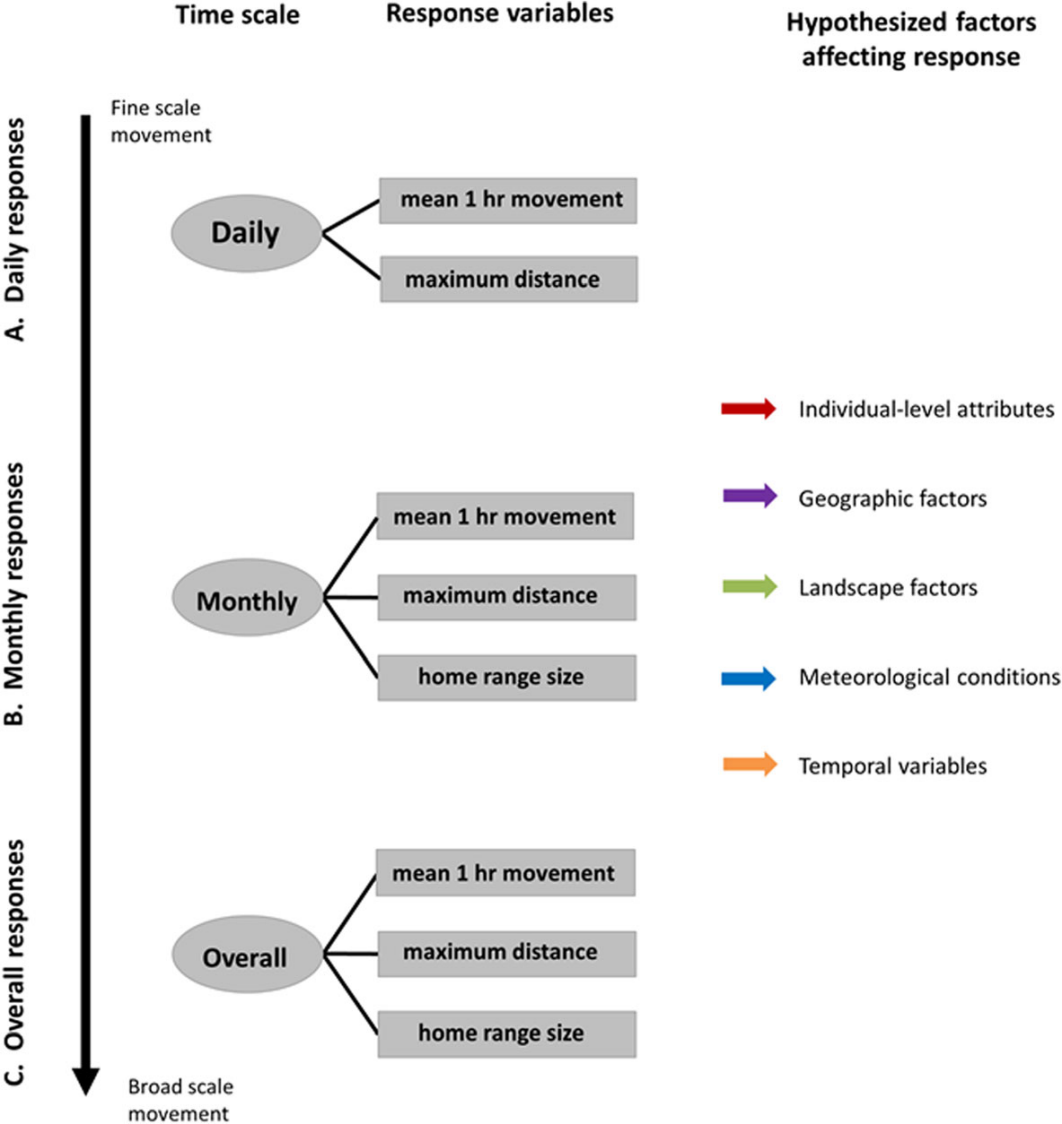
Schematic illustrating the three temporal scales (*circles; 1st column*) analyzed from wild pig location data in the southeastern U.S.A. from 2004 to 2016 along with which movement response variables were assessed at each temporal scale (*rectangles; 2nd column*). The *last column on the right* shows the five general categories (temporal, geographic, landscape, meteorological, and individual-level) affecting movement and home range size that were tested for each temporal scale

In addition to the movement metrics, factors affecting home range size at the monthly and overall scales were also explored (Fig. 2). Home range size estimates at the daily scale were not estimated due to limitations in sample size. To be consistent with previous literature, estimation of monthly and overall home range size was done with 95% minimum convex polygons (MCP) using the adehabitatHR package [49] in the statistical computing software R [50]. Effects of sample size (number of fixes) on home range size estimation at the overall scale is shown in Additional file 1: Figure S4D. Overall home range size was also estimated using autocorrelated kernel density estimation (i.e., akde in the ctmm package; [51, 52] to understand potential ramifications of using MCP for understanding drivers of home range size and for comparison to current and future literature. AKDE is a more modern method that accounts for spatial autocorrelation which is inherent in GPS data with frequent fixes (e.g., hourly), allowing for better projection of future home range use, and thus is thought to be more representative of home range use over a longer timescale [52]. In order to implement the AKDE method an appropriate autocorrelation structure must be identified within a continuous-time movement model (CTMM). We selected initial autocorrelation parameter estimates by visually assessing pooled population variograms for individuals within each study (i.e., variogram.fit in RStudio; [51]). We used a population approach based on locations within unique studies for choosing the intial autocorrelation structure since fix rates and individual behaviors were most consistent within studies. These parameter guesses were then used as the starting values for fitting the CTMM, from which AKDE estimates of home range size were obtained. We show differences in MCP versus AKDE estimates in Additional file 1: Figures S4A-C and S5.

### Predictor variables

#### Meteorological data

Daily-scale weather data were obtained by aggregating hourly values from the 1/8th degree meteorological forcing dataset for Phase 2 of the North American Land Data Assimilation System (NLDAS-2) [53, 54]. The observation-constrained meteorological variables of NLDAS-2 span 1979-present and are considered to be of suitable quality for use in biological and ecological modeling applications over North America [55]. Daily NLDAS-2 variables included in the analysis were maximum/minimum/mean air temperature (°C), mean relative humidity (%), mean saturation vapor pressure deficit (mm Hg), total precipitation (mm), mean surface pressure (Pa), mean wind speed (m s^−1^) and total growing degree days with respect to 10 °C (GDDs) (Additional file 1: Table S1). Daily data were then aggregated to monthly average maximum/minimum/mean temperature (°C) and monthly total precipitation (mm) for lags of up to 1 year. The monthly lagged data were included as proxies for biotic productivity to reflect seasonal fluctuations in environmental conditions, which could also be an indicator of resource availability. All environmental predictor variables were interpolated using the daily or monthly home range centroids (mean latitude and longitude) of the wild pig locations.

#### Landscape data

Distances from home range centroids to various resources including water bodies, streams, agricultural fields, and forest cover were compiled at the daily, monthly and overall scales (Additional file 1: Table S1). Distances to the nearest major, medium, and minor roads (e.g., interstates, highways, and unpaved roads) were also included. Several road classes were used to account for differences in traffic density and speed, as well as anthropogenic activity. All distance measurements were calculated in ArcGIS 10.3 [56].

#### Geographic data

Ecoregion types were also obtained for the daily, monthly, and overall scale wild pig home range centroids. There were nine unique level III ecoregion types in our study area including Southern Coastal Plain, Southeastern Plains, Mississippi Alluvial Plain, Western Gulf Coastal Plain, South Central Plains, Ozark Highlands, Central Irregular Plains, East Central Texas Plains, and Southern Texas Plains [57].

#### Individual-level attributes

Sex as well as age class information was available for most of the individuals included in our analyses. In addition, some of the original studies included management effects such as trapping, hunting, or harassment (chasing with dogs, helicopters, etc.) and therefore, we also explored the effects of management as an indicator variable in our analyses (Additional file 1: Table S1).

### Statistical analyses

We independently tested five broad categories of predictors for each response (MHD, MxD, and home range size) at each scale (daily, monthly, overall): individual-level attributes, meteorological conditions, temporal factors, geographic characteristics, and landscape-level factors (Fig. 2; Additional file 1: Table S1). We quantified the relationships of these five categories of predictor variables separately for each response at each scale using non-linear, parametric models that accounted for spatial dependencies among observations. We also explored the effects of management activities that were conducted in our individual studies, although none significantly affected movement rates on the scales we examined.

Since most of our meteorological variables were highly correlated, we used a two-step approach to modeling the effects of meteorological conditions (Additional file 1: Figure S6). We first employed random forest regression to initially screen predictor variables because of its ability to deal with large datasets, high-order interactions, and multicollinearity [58, 59]. Then, the top uncorrelated (*r* < 0.5) meteorological predictors for each response on each scale were carried forward into the parametric model for interpretation (Additional file 1: Figures S7-S10) since regression with many correlated variables may result in biased inference. However, in the meteorological models for MHD at the monthly scale and MxD at the overall scale, the GAMM models would not converge due to the particular combination of predictor variables. In these cases, we visually assessed the variable importance plots provided by the random forest regression (Additional file 1: Figures S9 and S10) to reduce the number of predictor variables, and included the top uncorrelated predictors in the GAMM models for which adding additional predictor variables did not significantly improve the predictive accuracy of the models (Additional file 1: Figure S6).

To quantify the effects of the covariates, we employed GAMMs [60] that allowed us to account for non-linear relationships between our responses and predictor variables, as well as caveats associated with our data. GAMMs can assume smooth, non-linear relationships between response and predictor variables by expanding the projection space of predictor variables through the use of basis functions [61–64]. In our analyses, we used a regularization approach to prevent overfitting models and employed penalized cubic regression splines with equally spaced knots for our smooth terms that shrink parameter estimates towards zero when they are less influential [60, 63, 65]. All meteorological covariates were non-linearly related to our response variables so they were included as smooth terms in all models while distance measures were considered to be linearly related.

Differences in temporal resolution between studies and individuals were accounted for in several ways depending on the scale of the data. In the daily scale models, we adjusted for precision of movement values by weighting by the number of locations per wild pig per day. We also accounted for repeated observations by the same individuals and variation among studies by specifying the wild pig and study identification factors as random effects. Because of strong autocorrelation at the daily scale, we included a linear autoregressive term in all daily-scale models. At the monthly scale, repeated observations and variation among studies were accounted for in the same way as in the daily models. However, the average number of locations each wild pig had per month were used as weighting factors to account for precision. In the overall scale models, study identification was used as a random effect while weighting was based on the average number of locations per day for each wild pig. To control for spatial correlation, we included latitude and longitude as smooth terms in all models.

## Results

### Descriptive statistics

Average MxD was between 0.80 km and 2.12 km with standard deviations ranging from 0.51 to 0.8 km, while average MHD was lower and varied from 0.35 to 0.42 km with corresponding standard deviations ranging from 0.34 to 0.51 km across temporal scales. The average monthly home range size (MCP method) was 3.4 km^2^ (sd = 4.6 km^2^) with a median of 1.8 km^2^ – indicating that most values were smaller than 3.4 km^2^. The overall average home range size was: 6.1 km^2^ (sd = 7.8 km^2^; MCP) and 12.4 (sd = 21.0 km^2^; AKDE); twice as high for AKDE relative to MCP (Additional file 1: Figure S4A). However, median overall home range sizes by both methods were comparable (3.4; MCP and 4.7; AKDE) and 70% of the estimates by both methods were within 2 km^2^, indicating that home range sizes are typically quite small by both methods (Additional file 1: Figure S4B, C). When AKDE and MCP were not in agreement, AKDE tended to estimate much higher home range sizes relative to MCP (up to ~95 km^2^ higher; Additional file 1: Figure S4A-C). Visual inspection of movement data showed: 1) for AKDE < MCP, longer movements were made on average and both metrics estimated relatively large home ranges (Additional file 1: Figure S5, middle column), 2) for AKDE > > MCP, average movements tended to be quite low but there appeared to be trends in time with movement (Fig. 5, right column), and 3) for AKDE ≈ MCP, movements tended to be low on average and there were no patterns in the magnitude of movement over time (Fig. 5, left column). Considering all the data, AKDE did not converge for 10 out of 227 individuals (4.4%), with total fixes ranging from 278 to 8281.

Missouri and Texas had the greatest average movement rates, while South Carolina had the lowest. Similarly, the average home range size was greatest in Missouri, but lowest in Florida (Additional file 1: Figure S2). Average movement rates were only slightly greater, but statistically insignificant, for individuals (or studies) that experienced some management effect (e.g., hunting, trapping, chasing with dogs, or other types of harassment) with a mean MxD and MHD of 0.81 and 0.43 km with management, and 0.79 and 0.41 km without management, respectively. Similarly, overall average home range sizes were also slightly greater with management (6.2 km^2^ for MCP and 15.6 km^2^ for AKDE) than without management (6.1 km^2^ for MCP and 11.2 km^2^ for AKDE).

### Maximum distance (MxD)

**Fig. 3:**
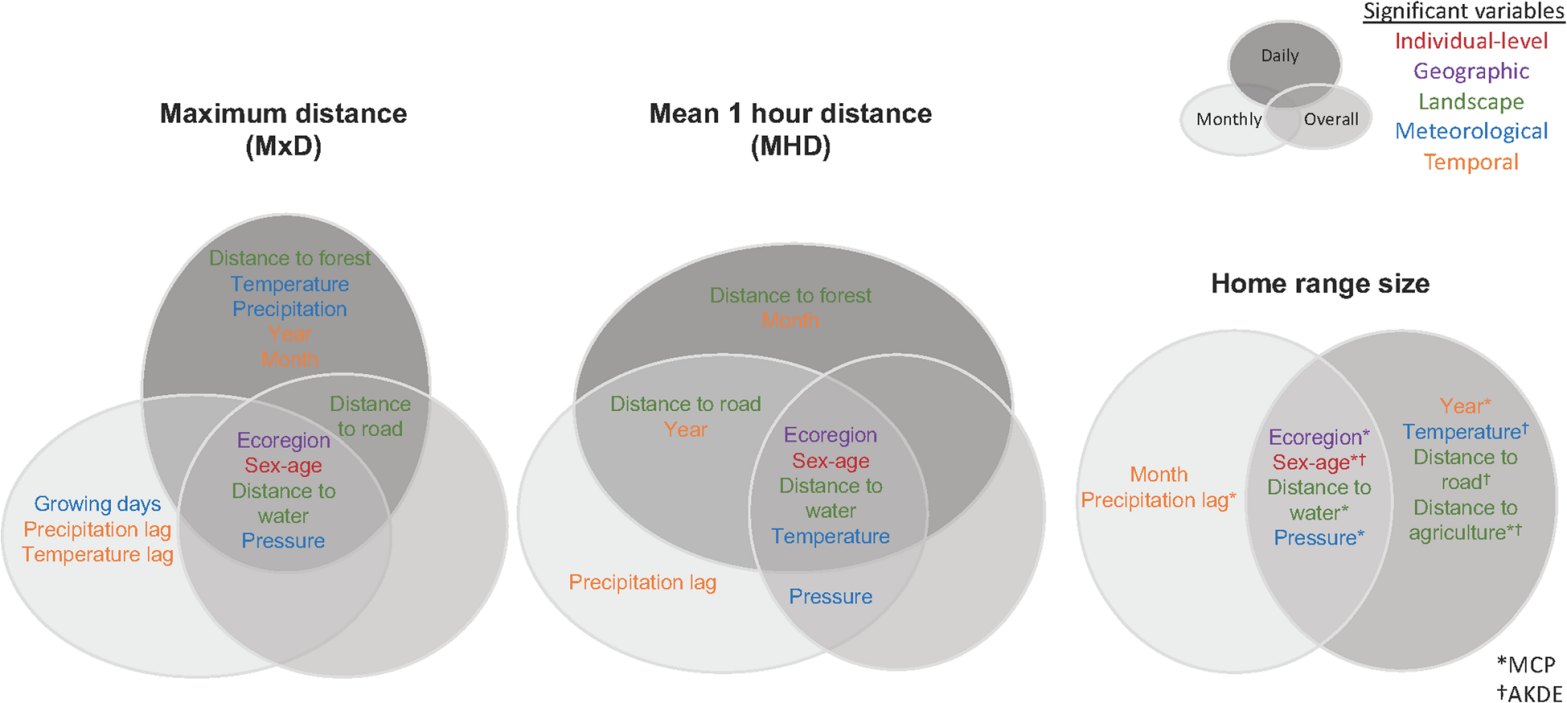
Venn diagrams which summarize the significant variables identified by the GAMM models for each response (MxD, MHD, and home range size) at each temporal scale (daily, monthly, and overall). The *top circle* for each movement response represents the daily scale, while the *bottom left* and *right circles* represent monthly and overall scales, respectively for movement responses and home range size. Variables are colored according to category tested where *red* represents individual-level attributes, *blue* represents meteorological variables, *purple* depicts geographic factors, *green* represents landscape variables, and *orange* depicts temporal factors. Factors contained in all *circles* (i.e., *top, left, and right*) such as ecoregion, sex-age, and distance to water, were significant in models across the three temporal scales

**Fig. 4:**
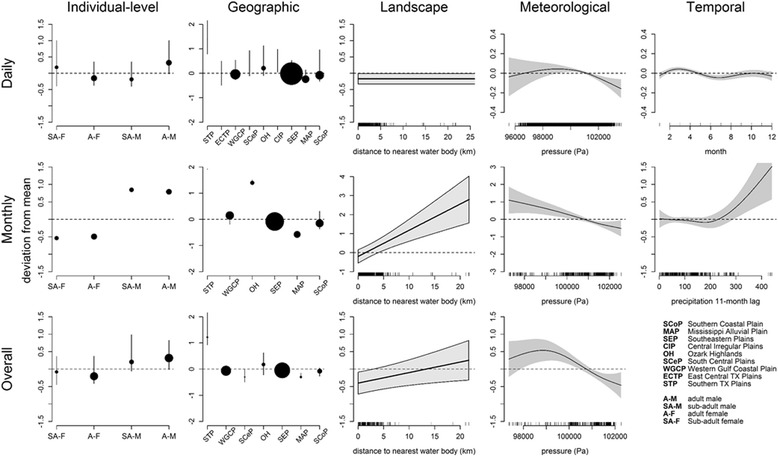
Relationships between significant variables for the average maximum daily distance (MxD) analyzed from wild pig location data in the southeastern U.S. from 2004 to 2016 across temporal scales. *Top row* depicts the daily scale, *middle row* shows the monthly scale, and *bottom row* depicts the overall scale. *Columns* correspond to the five broad categories of predictor variables tested

Individual-level attributes (sex and age), ecoregion, atmospheric surface pressure, and distance to the nearest water source were significantly associated with MxD across all temporal scales (Fig. 3). Both the shape and magnitude of the relationships of these predictors and movement differed across scales (Fig. 4). Males moved more than females at the monthly and overall scales (largest difference ~ 1.3 km), whereas sub-adult males did not move more than sub-adult females at the daily scale. Pressure displayed a similar, concave (i.e., upside-down “U”) pattern in MxD across daily and overall scales with above-average movement occurring between 97,000 and 101,250 Pa (28.6–29.9 Hg). However, at the monthly scale the relationship of pressure and movement was linear, indicating more movement at lower pressure (Fig. 4). In addition to pressure, temperature and precipitation were also significant in the meteorological model at the daily scale (R^2^ = 0.26), while average growing degree days was significant in the meteorological model at the monthly scale (R^2^ = 0.19). Like pressure, temperature at the daily scale also exhibited a concave relationship with MxD, while precipitation had a negative relationship with movement (Additional file 1: Figure S11). The average growing degree days at the monthly scale showed a weak, negative relationship with MxD (Additional file 1: Figure S11). At the monthly scale, only distance to the nearest water source was significant in the landscape model (R^2^ = 0.14), but had a greater impact on monthly and overall-scale movement than on daily movement (Fig. 4). Distance to the nearest road was also significant in the landscape models at the overall (R^2^ = 0.26) and daily scales (R^2^ = 0.27), and had a negative effect on MxD (Additional file 1: Figure S11). The landscape model for MxD at the daily scale also included a weak negative effect of distance to the nearest forest cover (Additional file 1: Figure S11). The variables year (Additional file 1: Figure S11) and month (Fig. 4) were significant in the temporal model at the daily scale (R^2^ = 0.1), where reduced movement occurred in the summer months. Lagged precipitation and lagged temperature were significant in the temporal GAMM models at the monthly scale (R^2^ = 0.22), where both had a generally positive effect on MxD (Fig. 4).

### Mean hourly distance (MHD)

**Fig. 5:**
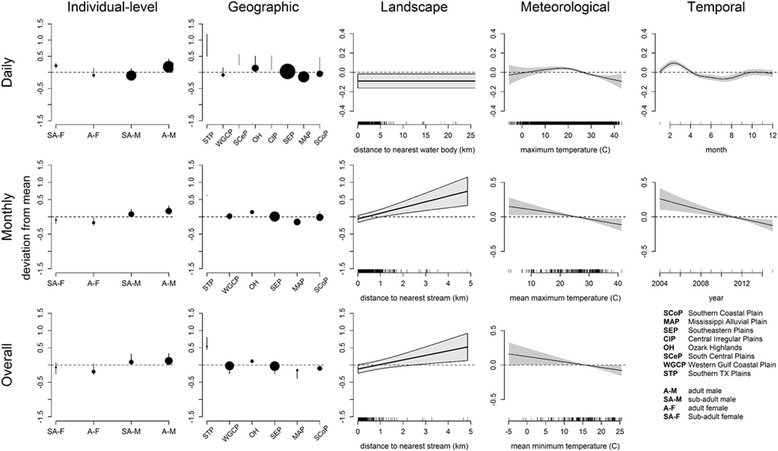
Relationships between significant variables for the average hourly distance moved (MHD) analyzed from wild pig location data in the southeastern U.S.A. from 2004 to 2016 across temporal scales. *Top row* depicts the daily scale, middle row shows the monthly scale, and *bottom row* depicts the overall scale. *Columns* correspond to the five broad categories of predictor variables tested

Similar to MxD, sex and age, ecoregion, and distance to the nearest water source were significant across all temporal scales for MHD, and the strength and shape of relationships between movement and predictors were dependent on temporal scale of movement (Fig. 3). Wild pigs in the Mississippi Alluvial Plain (MAP) ecoregion were found to have the lowest MHD, and wild pigs in the Southern Texas Plain were found to have the greatest MHD across all scales (Fig. 5). As with MxD, distance to the nearest water source was not as influential on MHD at the daily scale and a stronger, positive effect was detected with distance to the nearest stream at the monthly and overall scales (Fig. 3). Temperature was significant in the meteorological models at the daily (R^2^ = 0.14) and overall scales (R^2^ = 0.28), while average pressure was significant at the monthly (R^2^ = 0.13) and overall scales (Additional file 1: Figure S12). Comparable to MxD, temperature displayed a concave relationship with MHD at the daily scale, but was linearly related to movement at the monthly and overall scales (Fig. 5). In addition to distance to the nearest water sources, distance to the nearest road was significant and had a negative effect on movement in the landscape model for MHD at the monthly (R^2^ = 0.09) and overall (R^2^ = 0.18) scales (Additional file 1: Figure S12). Distance to the nearest forest cover was also significant and had a negative effect on MHD in the landscape model at the daily scale (R^2^ = 0.15; Additional file 1: Figure S12). Year was significant in both temporal models at the daily (R^2^ = 0.1) and monthly (R^2^ = 0.16) scales where below-average MHD was predicted for summer months (Fig. 5 and Additional file 1: Figure S12). Precipitation with a lag was also significant at the monthly scale (Additional file 1: Figure S12), where more movement was predicted when there was more precipitation several months prior.

### Home range size

**Fig. 6:**
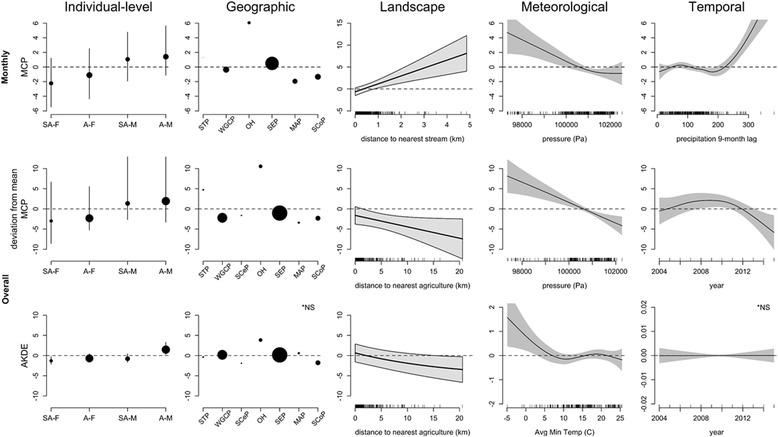
Relationships between significant variables for home range size analyzed from wild pig location data in the southeastern U.S.A. from 2004 to 2016 across temporal scales where the *top row* depicts the monthly scale and the *middle* and *bottom rows* depict the overall scale for MCP and AKDE estimates of home range size, respectively. *Columns* correspond to the five broad categories of predictor variables tested. *NS indicates variables that were not significant with AKDE home range values

Significant predictors of home range size across monthly and overall scales were similar to the movement responses, where sex and age, ecoregion, and distance to the nearest water source or agriculture were significant (Figs. 3 and 6). As with the movement responses, male wild pigs had home ranges 3.5—5 km^2^ larger than females (Fig. 6). Smaller home ranges were found in the Mississippi Alluvial Plain (MAP), Southern Coastal Plains (SCoP), Western Gulf Coastal Plains (WGCP) and Southeastern Plains (SEP) ecoregions relative to those found in the Ozark Highlands (OH) ecoregion (Fig. 6). Pressure was the only significant meteorological variable at both scales using MCP, while temperature was the only significant meteorological variable using AKDE. Similar to other movement response variables, pressure and temperature were negatively related to home range size, and did not show a concave relationship as with daily scale metrics of movement (Fig. 6) (although the relationship of AKDE home ranges and temperature was non-linear). Distance to the nearest water source was positively related with home range size at both scales (Additional file 1: Figure S13), although it was not significant using the AKDE estimates. Distance to the nearest agricultural field was significantly negative and linear at the overall scale only, for both MCP and AKDE estimates, although the relationship was weaker for AKDE estimates (Fig. 6). The variables month and precipitation with a lag were significant at the monthly scale (Additional file 1: Figure S13), while year was significant at the overall scale for MCP but not AKDE home range size estimates (Fig. 6).

## Discussion

Drivers of movement capacity may differ in shape and magnitude depending on the temporal scale of movement and spatial extent of the data. Understanding spatial differences in reaction norms could be important because driving factors may constrain movement differently across space and time. For example, we found a distinct movement reaction norm in response to temperature, but our data were limited to the southern U.S.A. Wild pigs occurring in colder climates may not display the same constraints or reaction norm shape. Elucidating these relationships across a wide geographic gradient will help us understand which factors driving movement are primarily a result of physiological constraints, and which factors affecting behavior may be driven by surrounding landscape characteristics.

The temporal scale of movement analyzed should coincide with the specific research questions, and the covariates used in the analyses should complement processes that act on the same temporal scale. For example, reproduction in some wildlife species is thought to affect home range sizes around the time of birth [45], but this phenomenon could be overlooked at certain scales. In fact, one sow had a monthly home range size half as large as usual when she gave birth, but this could not be determined at the overall scale. Thus, our results emphasize the importance of choosing the correct spatial and temporal scales for conducting analyses aimed at predicting movement due to climate or other factors.

We also found evidence of regional differences in the magnitude of various factors affecting movement capacity, such as proximity to water resources, which reflect locally-adapted behaviors. For example, the slope on the linear relationship between MHD and distance to the nearest water body is twice as large for wild pigs in one area of Texas (β = 0.32) as it is for wild pigs in an area of Florida (β = 0.14). This difference is likely because water resources are less prominent in Texas than in Florida so wild pig movement in Texas is more affected by distance to the nearest water body. Conversely, if the data from Georgia or Louisiana are analyzed individually, no significant effect of distance to the nearest water body is detected because there are abundant water resources available (i.e., no wild pigs in our study from Georgia or Louisiana were more than 1.05 km away from a stream or water body).

Furthermore, general patterns in movement responses can be determined from pooling and analyzing data from multiple sources, so that common relationships between variables affecting movement can be quantified. Additionally, these patterns in behavior can be predicted for different areas, and used to provide guidelines for management purposes. Due to the resource limitations of many studies, the statistical power necessary to identify certain factors affecting movement capacity may be limited because of small sample sizes. In many cases, GPS collars malfunction or fall off the animal prematurely, resulting in unbalanced statistical designs. In other cases, there may not be sufficient resources available to monitor more than a handful of individuals over a short period of time so examining seasonal effects on movement capacity can be challenging. Thus, general inference can be acquired on mechanisms affecting wildlife species across broad spatial and temporal scales with an approach similar to what we present here, which is useful for developing optimal strategies for managing or conserving species.

### Meteorological effects

Pressure was highly influential to both movement responses and home range size across all temporal scales, which has been observed in other species including deer [41], red fox [37], coyote [42], domestic cattle [40], and moose [38]. The concave relationship between pressure and MxD at the daily scale suggests an optimal range for pig movement (97500–101,250 Pa), which corroborates previous work where increased wild pig activity has been observed when pressure is high than low, typically preceding a frontal boundary (Martin J. Factors Affecting Hog Movement. USDA. personal communication). Similarly, [66] found a behavioral response in mountain sheep with changing pressure, conceivably in anticipation of an extreme weather event. Likewise, the correlation with monthly home range size and pressure could be a result of seasonal variation in weather patterns. At the daily scale, very low pressures reduced movement rates (i.e., likely during a storm), whereas at the monthly scale, low pressure systems from storms were averaged across the month and thus the concave relationship was too weak to observe (and was not even significant using the AKDE method of estimation). This implies that using monthly or longer meterological averages may not be a good predictor of changes in animal movement for applications such as determining optimal baiting conditions, or predicting impacts of climate change, which could affect movement on finer spatial scales. Nonetheless, using easy-to-obtain meteorological variables such as pressure on small spatial scales to summarize severe storm conditions can help determine how movement behaviors are affected by extreme weather.

Temperature also appeared to be highly influential on wild pig movement across all scales where movement was reduced during extreme conditions. Similar to pressure, the relationship between temperature and movement at the daily scale was concave, whereas a linear (or non-concave, non-linear for AKDE) relationship was found at broader scales – thus not only the magnitude but also the shape of the reaction norm changed depending on temporal scale. Since there is a consistent pattern in the shape of relationships between movement rates and our meteorological variables, these relationships may in fact reflect seasonal weather fluctuations. For example, it has been suggested that wild pig behavior is primarily nocturnal during the summer months, but tends to be more diurnal during the fall, winter, and spring months [45, 67]. Coyotes have also been shown to have seasonal variation in home range size as a result of fluctuations in resource availability and behavior [68]. Quantifying the magnitude and shape of these reaction norms is important for predicting constraints in animal movement capacity due to climate change or invasion into new geographic areas.

### Temporal effects

In addition to current meteorological conditions, we tested the effects of lagged meteorological conditions on movement assuming that weather conditions in the past may determine current food abundance due to seasonality or interannual variation in weather patterns. Examining seasonal effects this way as opposed to using month as a categorical variable was more informative because of the large geographical scale we examined – e.g., January in Florida is not the same as January in Missouri. We found relationships between the 2-month temperature and 9-month precipitation lag variables and monthly home range size, which could reflect seasonal differences in movement patterns resulting from interannual fluctuations in food abundance. Our results are consistent with [44, 69, 70] which found evidence of range shifts in wild pigs due to fluctuations in resource availability arising from seasonal changes, such as migration towards agricultural areas during the summer months. Other studies have also found seasonality to significantly affect home range size of species such as coyote, raccoon, mule deer, and elk as a result of food availability [43, 68, 71, 72]. Thus, lagged meteorological variables may be a useful means of quantifying seasonal effects across large spatial scales which show different weather patterns throughout the year.

### Landscape effects

We found that distances to various resources, especially water, roads or agriculture, significantly affected movement rates across all scales, but the magnitude of this effect was greater at the monthly and overall scales. Many wild pigs in our analyses were likely near surface water such as puddles, small marshes, and vernal pools, and hence may not have been affected on a daily basis by the need for more substantial bodies of water. However, water bodies such as ponds, lakes, and rivers may be utilized more diurnally and thus their effect may only be reflected across longer time frames as in our monthly and overall scales.

Distance to the nearest major road was also found to affect wild pig movement across all scales. Roads can negatively affect wildlife species by creating barriers to movement [73]; however, the spread of invasive species can also be facilitated by roads [74]. Additionally, many vehicle collisions with wild pigs and other wildlife occur on roadways [75–78]. Therefore, studying the effects of roads as barriers or facilitators to animal movement will continue to be a critical component of wildlife conservation and will help reduce the risk of human-wildlife conflict.

Wild pigs are known to commonly exploit cropland, causing high monetary losses [16]. However, home range size, using both MCP and AKDE estimates, was found to have a negative, linear relationship with distance to the nearest agriculture predicting lower movement rates for wild pigs residing further away from agricultural resources. As most pigs were within 5 km of agriculture in our dataset (i.e., within reasonable distance to travel regularly to crops for feeding), the negative relationship suggests that pigs residing further away from crops, may not travel to the crops routinely (as we would expect a positive relationship in that case), and instead be using another food resource [70, 79, 80]. Our results also suggest that crops may influence wild pig movement ecology – pigs residing near crops, a massively abundant food resource, move less than those living further away and using different food sources.

### Geographic effects

Ecoregion was a significant factor in all models and across all scales, demonstrating the predictive utility of understanding the effects of broad geographic differences on movement capacity. Wild pigs in drier ecoregions (Southern Texas Plains and Ozark Highlands) had above-average movement rates across all scales, while pigs in mesic ecoregions (Mississippi Alluvial Plain and Southern Coastal Plain) had below-average movement rates. Wild pigs in the drier ecoregions are predicted to move more because water resources are further away on average. Thus, ecoregion may describe numerous characteristics of a landscape, and could be a simple predictor of movement levels which is valuable for planning management programs across a large spatial scale [81, 82].

### Individual-level effects

Sex and age had a significant effect on wild pig home range size and movement rates across all temporal scales. Unsurprisingly, adult male wild pigs were found to have larger home range sizes and greater movement rates than female pigs [31, 45, 83], which has also been observed in many other species such as raccoons [72, 84], bobcats [85], and moose [86]. Thus, individual-level attributes such as sex and age reflect social behaviors that affect movement and are important to consider for prediction and management. For example, female wild pigs are known to reside with other females and therefore large traps are more effective for capturing several females at the same time [87]. However, male wild pigs are known to be solitary, so hunting with dogs has found to be more effective at capturing males than females [45].

### Effects of MCP versus AKDE home range estimates

Our comparison of MCP and AKDE estimates showed that MCP captured a similar home range estimate as AKDE 70% of the time, but otherwise AKDE led to extremely high (up to 900% higher than MCP) or slightly lower (up to 36% lower than MCP) estimates. When AKDE < MCP, movements and home range sizes tended to be much larger for both estimates, relative to when MCP = AKDE or when AKDE > > MCP. When AKDE > > MCP, movements were lower on average but there were trends over time in maximum movement, which could indicate dispersal-type (non-stationary) behavior. The AKDE method we applied is based on the assumption that movement patterns are consistent in time such that these large estimates of home range using AKDE could be biased. When the movement data are non-stationary, the underlying movement model of AKDE anticipates more long-range movements in the future leading to a larger home range estimate. In addition, although AKDE is more statistically sound than MCP [52], the very high AKDE estimates do not match the trends seen in our movement metrics (MxD and MHD) at the overall scale (where MAP ecoregion individuals moved less relative to other ecoregions as MCP predicts), and they are large outliers relative to the general patterns of home range size, further supporting the idea that the movements in these individuals were not captured well by the model, producing biased estimates.

The large, outlier AKDE estimates also likely explain some of the differences in significance of predictors of MCP versus AKDE home ranges. For example, with the AKDE method, the ecoregion effect was removed and individuals in the MAP did not show lower than average home ranges as they did with MCP. However, this discrepancy was likely because of a large estimate for one individual. Specifically, considering all estimates from the MAP, the AKDE did not converge on an estimate for 3 individuals in the MAP (23% of the MAP data), was very close to MCP for 8 individuals (61.5% of the MAP data) but estimated a home range size 50 times higher for one individual (7.7% of the MAP data). Thus, some of the discrepancies among MCP and AKDE home range estimates in terms of the significance of predictors could be due to the much larger variation in AKDE versus MCP; specifically the very high estimates which may have been biased.

### Caveats and considerations

Using metrics such as maximum distance and home range size are beneficial because they can be calculated from a wide variety of temporal sampling schemes, and are commonly used making comparison with previous studies easier. On the other hand, metrics like MHD are more data-limiting because resolution can only be as fine as the most temporally sparse dataset. Furthermore, there were many times for which GPS fixes could not be obtained, or where fixes were insufficiently accurate to be included (e.g., had high dilution of precision). Aside from temporal resolution of telemetry data, there may be other discrepancies between studies which must be considered such as length and timing of monitoring individuals, which we accommodated using weights. We were unable to include all top predictor variables in two of our models (overall meteorological MxD and monthly temporal MHD) due to convergence issues. Therefore, for these models we only included variables in the subsequent GAMMs that highly affected the predictive accuracy in the random forest regression. One could also use a stepwise approach to choose an optimal set of predictor variables, or use alternative spline types or larger penalties in the GAMM model if convergence problems are encountered. However, a stepwise selection procedure may be computationally intensive, especially if there are a large number of data points, whereas a computationally efficient and non-parametric variable selection procedure such as random forests can be utilized without the need for extensive knowledge of spline regression. Therefore, our approach allowed for general inference of wild pig movement across the southern U.S.A. by including data from several different studies while accounting for differences in their designs and addressing issues with multicollinearity.

For simplicity of presentation, we did not look at interactions between extrinsic and intrinsic factors in our analysis. However, it is possible that extrinsic factors such as weather could result in different movement reaction norms as a function of individual-level attributes. For example, perhaps adult males show a different reaction norm to temperature relative to females such as [81] found, where home range sizes of males were not affected by drought conditions but female home range sizes changed according to food availability and temperature constraints. Quantifying these individual-based differences in reaction norms could improve our fundamental understanding of movement capacity and lead to better prediction of how meteorological changes may impact population ecology.

## Conclusions

Our analyses show how simple metrics can be used to quantify movement capacity across multiple spatio-temporal scales. Numerous challenges exist in combining multiple datasets, but these can be accounted for with a flexible modeling approach such as machine-learning algorithms and/or generalized additive models. Since the ecological community will inevitably continue collecting large telemetry datasets due to the increased availability of GPS technology, further analytical research could include developing novel methods to explicitly address challenges associated with using data from many different sources with varying degrees of resolution. Nonetheless, our approach is straightforward and easily implemented using existing packages in R with commonly known syntax, making it computationally feasible to apply to numerous temporal scales of data (see SI code). Therefore, similar to [1], we advocate for more cross-scale studies which inherently examine different components of movement across multiple scales within a single framework. While it is always important to focus data collection on an appropriate spatio-temporal scale for a target question, understanding cross-scale effects can provide additional insights that may help movement prediction as environments change. Also, movement constraints at one scale may be affected by conditions at another scale, generating different reaction norms for movement depending on the cross-scale context. To achieve the goal of increasing the frequency of cross-scale movement studies, we encourage the collaboration between wildlife researchers and managers by sharing data directly or through open-source databases (e.g., EURODEER, EUROBOAR, MoveBank, etc.).

## Additional files

Additional file 1: Additional figures. (PDF 2668 kb)

Additional file 2: Raw data and R code. (ZIP 2500 kb)
