# Supplementary material for: Quantifying drivers of wild pig movement across multiple spatial and temporal scales
Source: Mov Ecol. 2017 Jun 15;5:14. doi: 10.1186/s40462-017-0105-1 (PMC5471724; doi:10.1186/s40462-017-0105-1)
Supplement: Supplementary file 1 — Additional figures. (PDF 2668 kb) [file 40462_2017_105_MOESM1_ESM.pdf]

Supporting Information for:

"Quantifying drivers of wild pig movement across multiple spatial and temporal scales"

Shannon L. Kay, Justin W. Fischer, Andrew J. Monaghan, James C. Beasley, Raoul Boughton, Tyler A. Campbell, Susan M. Cooper, Steve S. Ditchkoff, Steve B. Hartley, John C. Kilgo, Samantha M. Wisely, A.Christy Wyckoff, Kurt C. VerCauteren, and Kim M. Pepin

Additional figures and details of data processing

Table S1. Predictor variables of individual-level attributes as well as meteorological, temporal, geographic, and landscape data investigated for each of the three responses. Temporal scales that included the predictor variable are listed in the left column.

	**Variable**	**Data Source**
**Individual-level**	sex.age (juvenile female, sub-adult female, adult female, sub-adult male, adult male)	Study-specific
**Meteorological Data**	air temperature (maximum, minimum, average) (°C)	1/8th degree meteorological forcing dataset for Phase 2 of the North American Land Data Assimilation System (NLDAS-2)
	total precipitation (daily total, monthly total) (mm)	
	mean surface pressure (Pa)	
	mean wind speed (m/s)	
	mean relative humidity (%)	
	saturation (mm Hg)	
	growing degree days (no units)	
**Temporal**	year	Study-specific
	month	Study-specific
	average monthly temperature lagged 1-12 months (mm)	NLDAS-2
	total monthly precipitation lagged 1-12 months (mm)	
**Geographic Data**	ecoregion	DATA.GOV; U.S. EPA
**Landscape Data**	distance to nearest stream (km)	USGS; NHD Flowline Medium Resolution
	distance to nearest water body (km)	USGS; NHD Waterbody Medium Resolution
	distance to nearest forested area (km)	NLCD 2011
	distance to nearest agricultural field (km)	NLCD 2011
	distance to nearest minor road (km)	HERE 2014 Q2 Street Network
	distance to nearest medium road (km)	HERE 2014 Q2 Street Network
	distance to nearest major road (km)	HERE 2014 Q2 Street Network
**Inherent attributes related to study**	number or average number of locations	Study-specific
	pigID	Study-specific
	study	Study-specific

**design** (included in every model)	management (indicator for whether or not individual/study was subject to management effects such as aerial gunning)	Study-specific
	latitude (°)	Study-specific
	longitude (°)	Study-specific

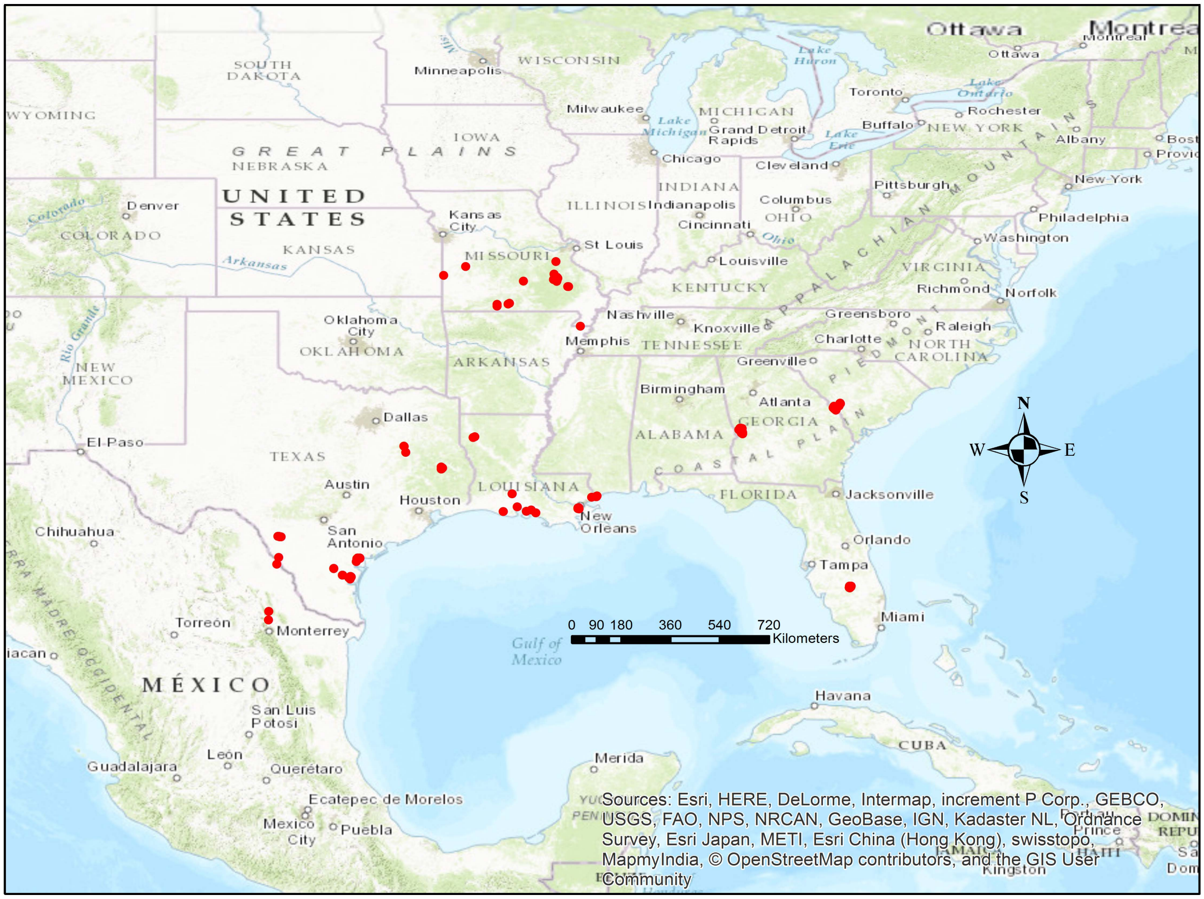

Figure S1. Map of study area where red dots depict home range centroids (mean latitude and longitude) of individuals used in meta-analysis.

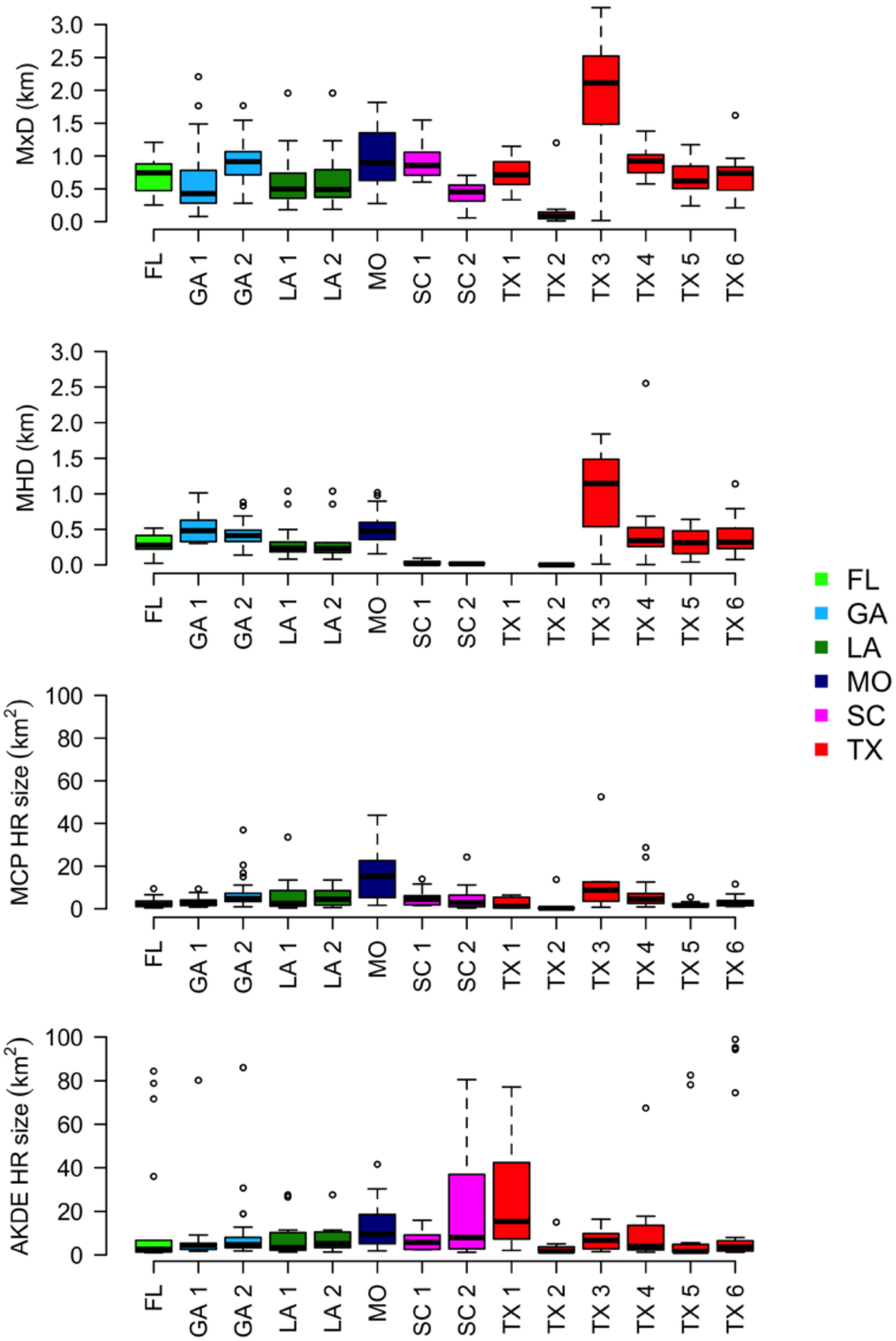

Figure S2. Response variables at the overall scale by study, colored by state. Top plot depicts the maximum distance response (MxD), the second plot shows mean distance moved in one hour (MHD), the third plot is home range size using MCP, and the bottom plot depicts home range size using AKDE.

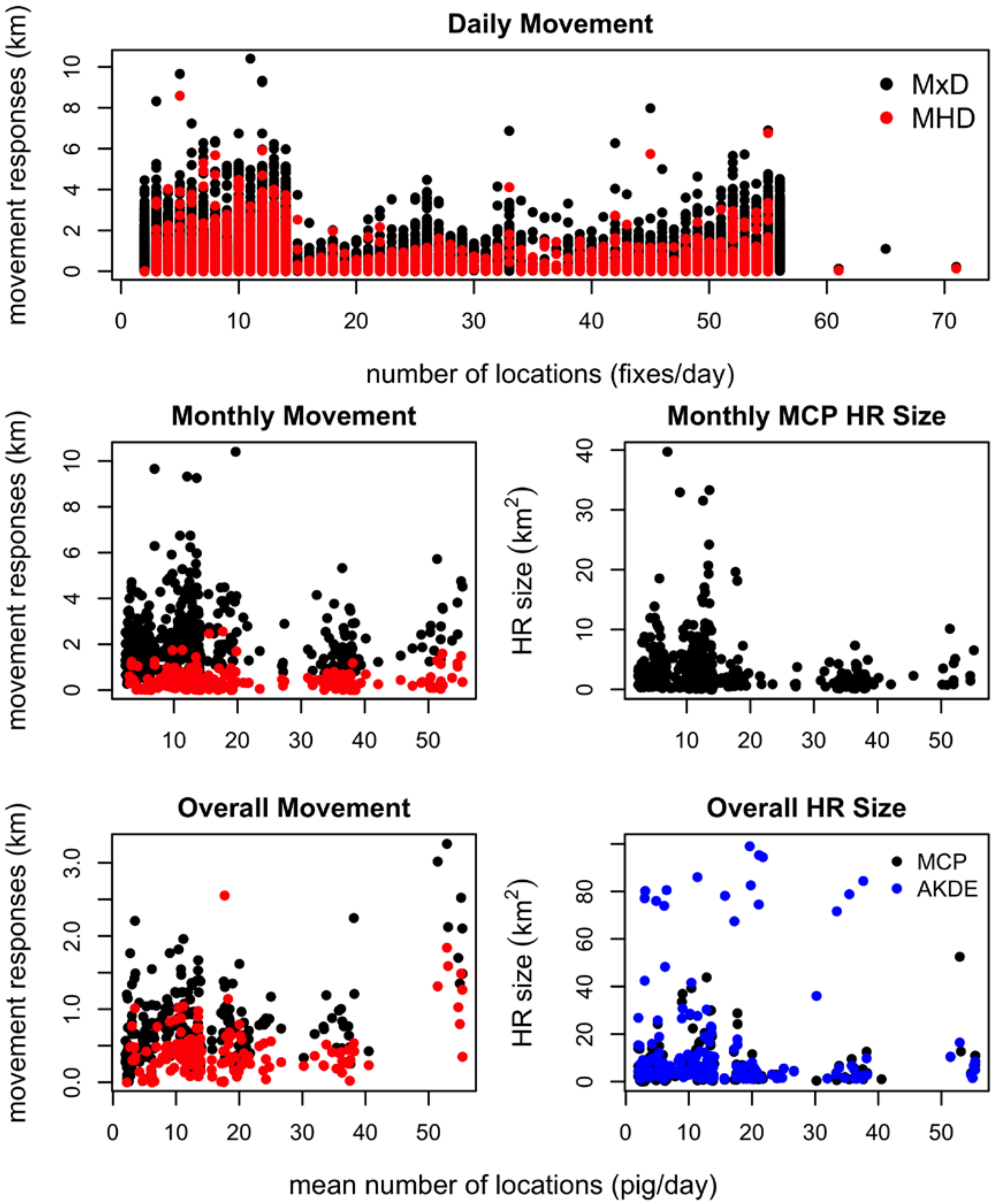

Figure S3. Response variables at daily (top), monthly (middle), and overall (bottom) scales by of number of locations and average number of locations per day showing potential sampling bias as a result of fix rate. Black and red dots show MxD and MHD movement responses, respectively, in the top panel and left column. Black dots in the right column depict home range size using MCP, blue dots show home range size using AKDE (overall scale only).

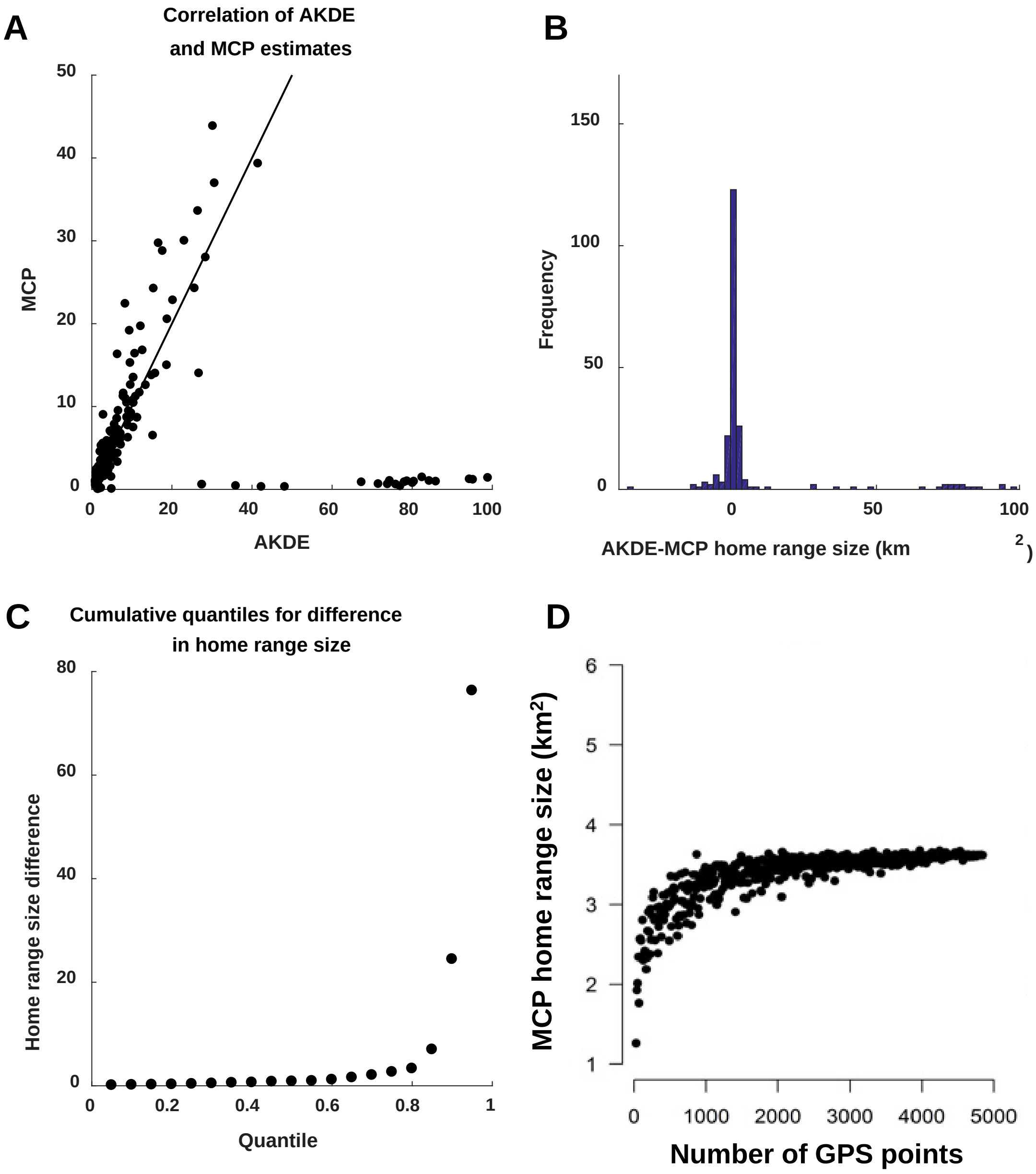

Figure S4. Estimates of home range size (km^2) using the minimum convex polygon (MCP) method versus an autocorrelation kernel density method (AKDE). A) Scatterplot of home range size estimates by both methods at the overall scale. The line shows the 1:1 relationship. B) Histogram of the difference between AKDE-MCP home range size estimates (using a 2 km bin size). C) Cumulative quantiles for the absolute difference in home range size between AKDE and MCP estimates. 70% of differences are less than 2 km. D) Estimates of overall home range size for one individual using MCP by the number of randomly selected relocations indicates an asymptote at 750-1000 locations. Thus, estimates based on fewer locations could be biased low.

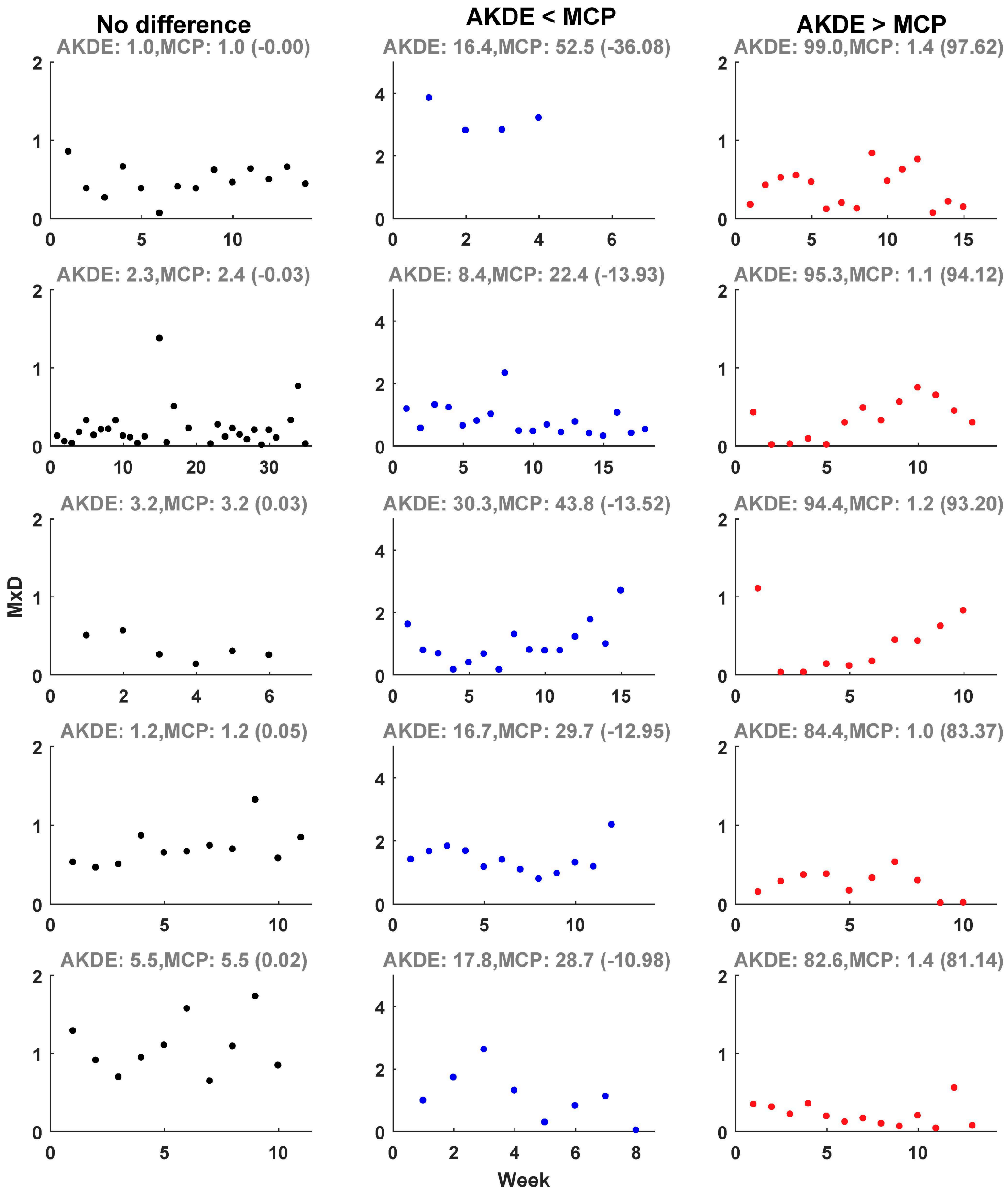

Figure S5. Weekly MxD over time for 15 individuals. Left column data are for the individuals whose MCP and AKDE estimates were least different (within 0.05 km^2). Middle column shows examples of individuals whose MCP was > AKDE. Right column shows individuals whose MCP was < AKDE.

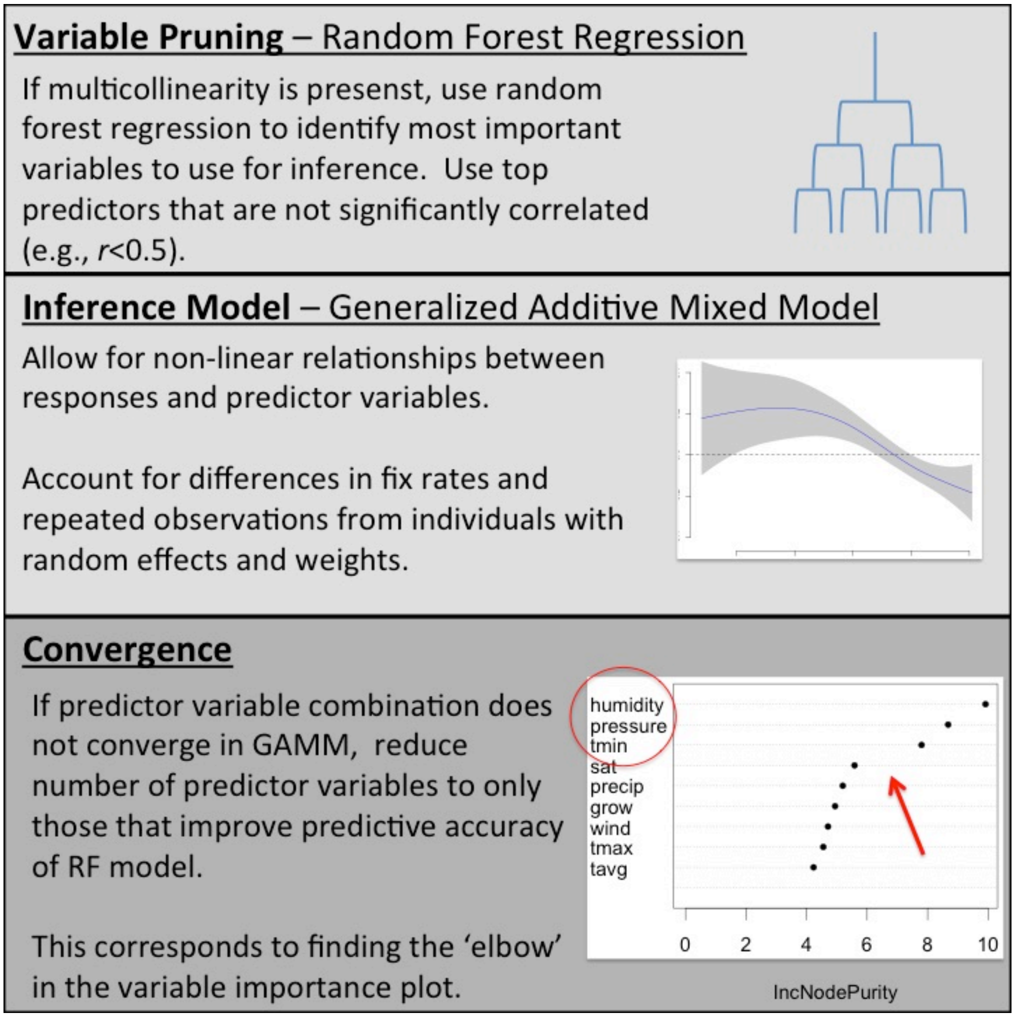

Figure S6. Description of model selection procedure for highly correlated datasets (meteorological models at all scales and temporal models at monthly scale). Top panel shows initial random forest regression of response variable and correlated predictor variables. Top variables without considerable linear correlation (*r*<0.5) in order of importance to predictive accuracy were taken into the GAMM model for inference (middle panel). Some correlation was acceptable at this stage due to regularization applied in the smooth terms of the GAMM. If convergence issues were present, the set of predictors was reduced to only the top predictors that did not substantially improve the predictive accuracy of the random forest regression (bottom panel).

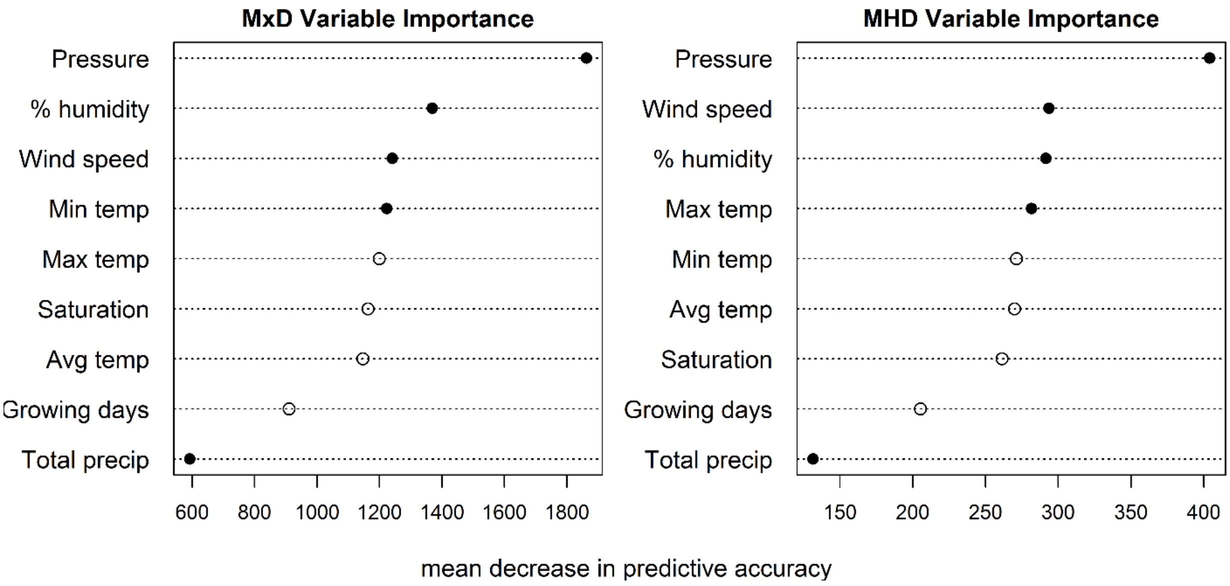

Fig S7. Variable importance plot from random forest regression analyses on meteorological variables at the daily scale where variables on the y-axis are ordered from most important (top) to least important (bottom). The x-axis shows the mean decrease in predictive accuracy (mean square error) when randomly permuting values of variables in the random forest. Variables in bold were carried into the GAMM models.

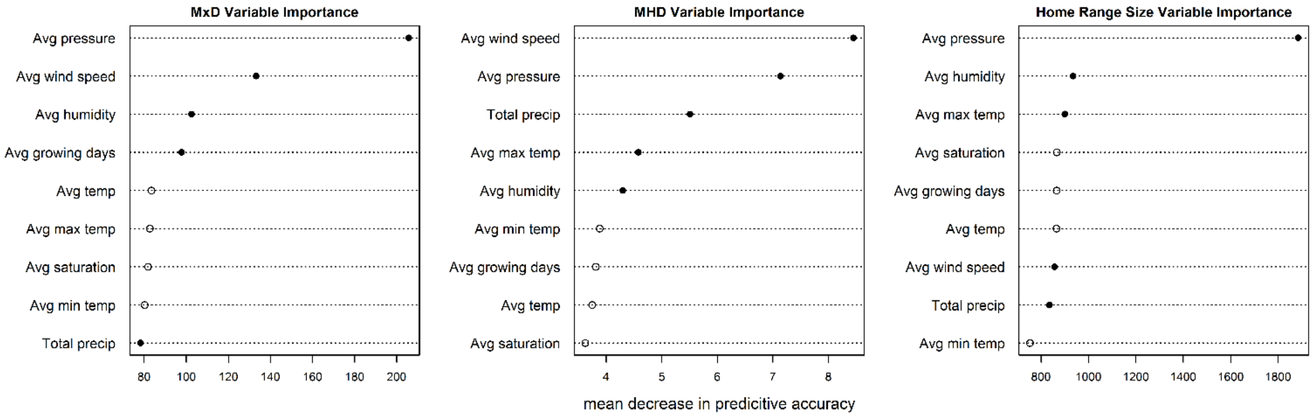

Fig S8. Variable importance plot from random forest regression analyses on meteorological variables at the monthly scale where variables on the y-axis are ordered from most important (top) to least important (bottom). The x-axis shows the mean decrease in predictive accuracy (mean square error) when randomly permuting values of variables in the random forest. Variables in bold were carried into the GAMM models.

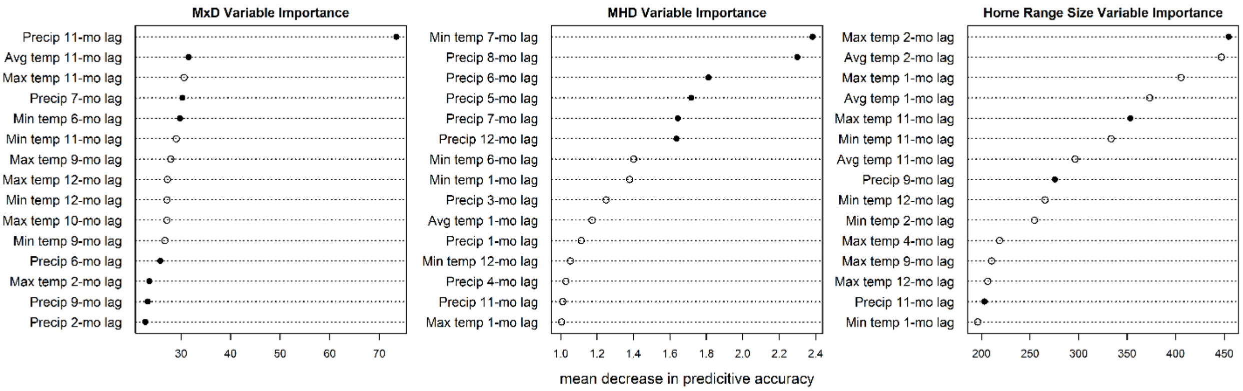

Fig S9. Variable importance plot from random forest regression analyses on temporal meteorological variables at the monthly scale where variables on the y-axis are ordered from most important (top) to least important (bottom). The x-axis shows the mean decrease in predictive accuracy (mean square error) when randomly permuting values of variables in the random forest. Variables in bold were carried into the GAMM models.

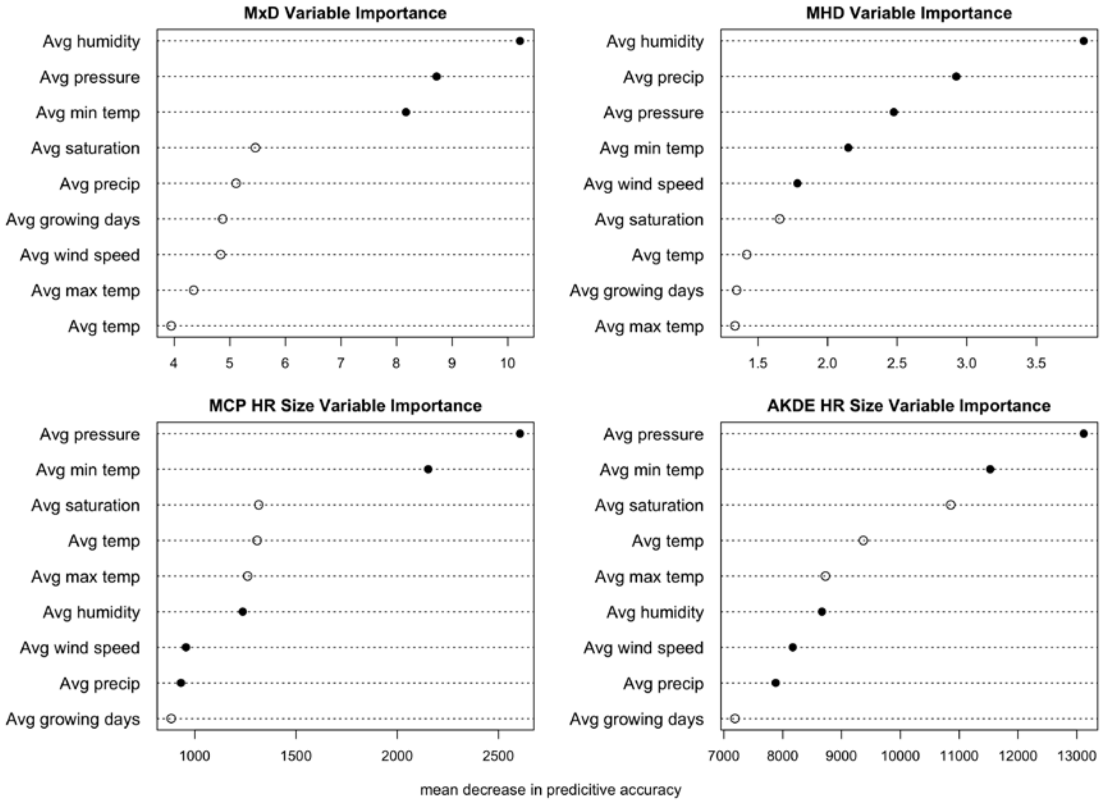

Fig S10. Variable importance plot from random forest regression analyses on meteorological variables at the overall scale for MCP and AKDE home range size estimates. Variables on the y-axis are ordered from most important (top) to least important (bottom). The x-axis shows the mean decrease in predictive accuracy (mean square error) when randomly permuting values of variables in the random forest. Variables in bold were carried into the GAMM models.

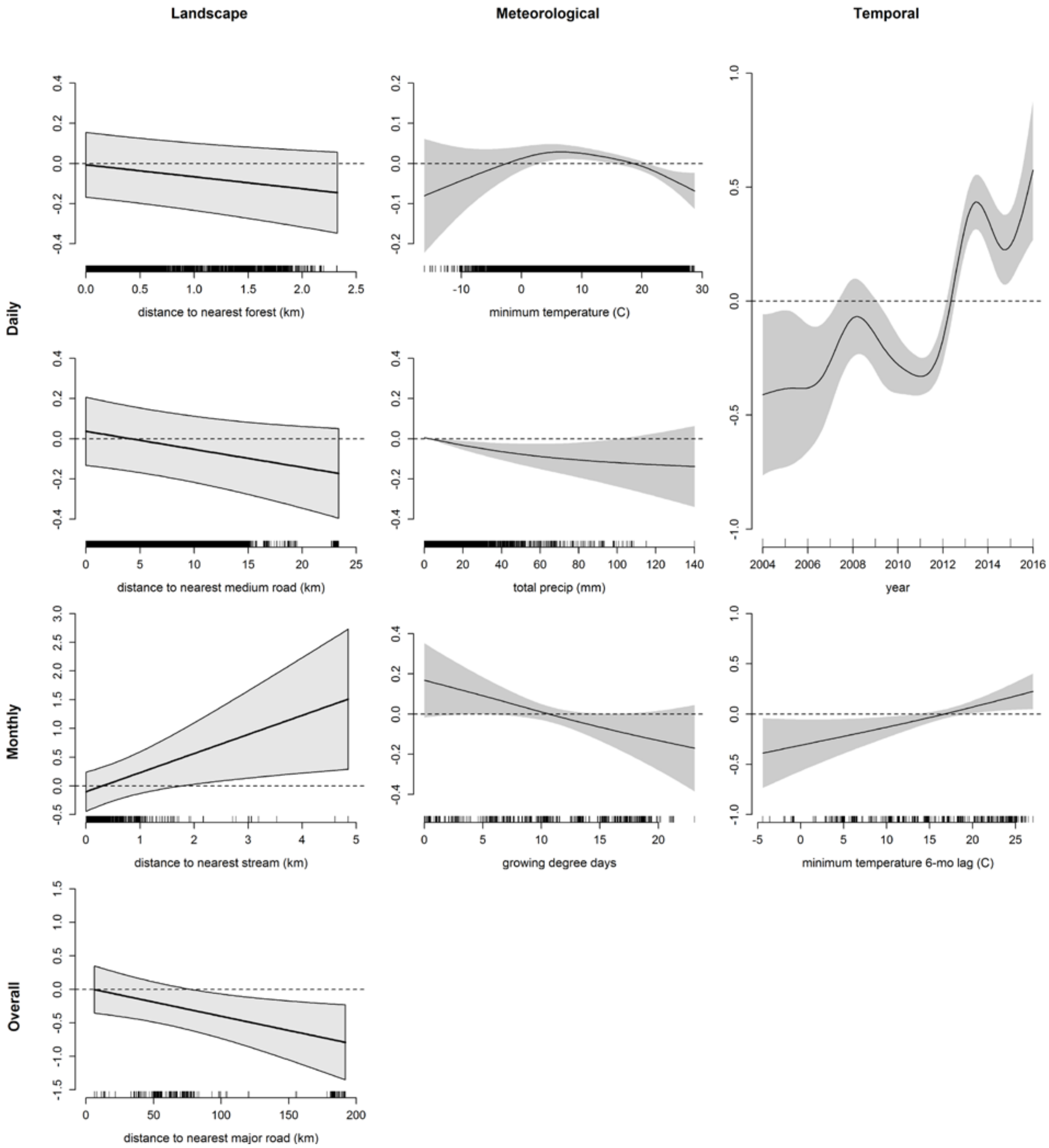

Figure S11. Relationships between significant variables not shown in the main text for the average maximum daily distance (MxD) analyzed from wild pig location data in the southeastern U.S.A. from 2004—2016 across temporal scales. Top row depicts the daily scale, middle row shows the monthly scale, and bottom row depicts the overall scale. Columns correspond to the broad categories of predictor variables tested. The x-axes display individual variables tested while the y-axes show the deviation from the average MxD. The dashed horizontal line corresponds to the average MxD.

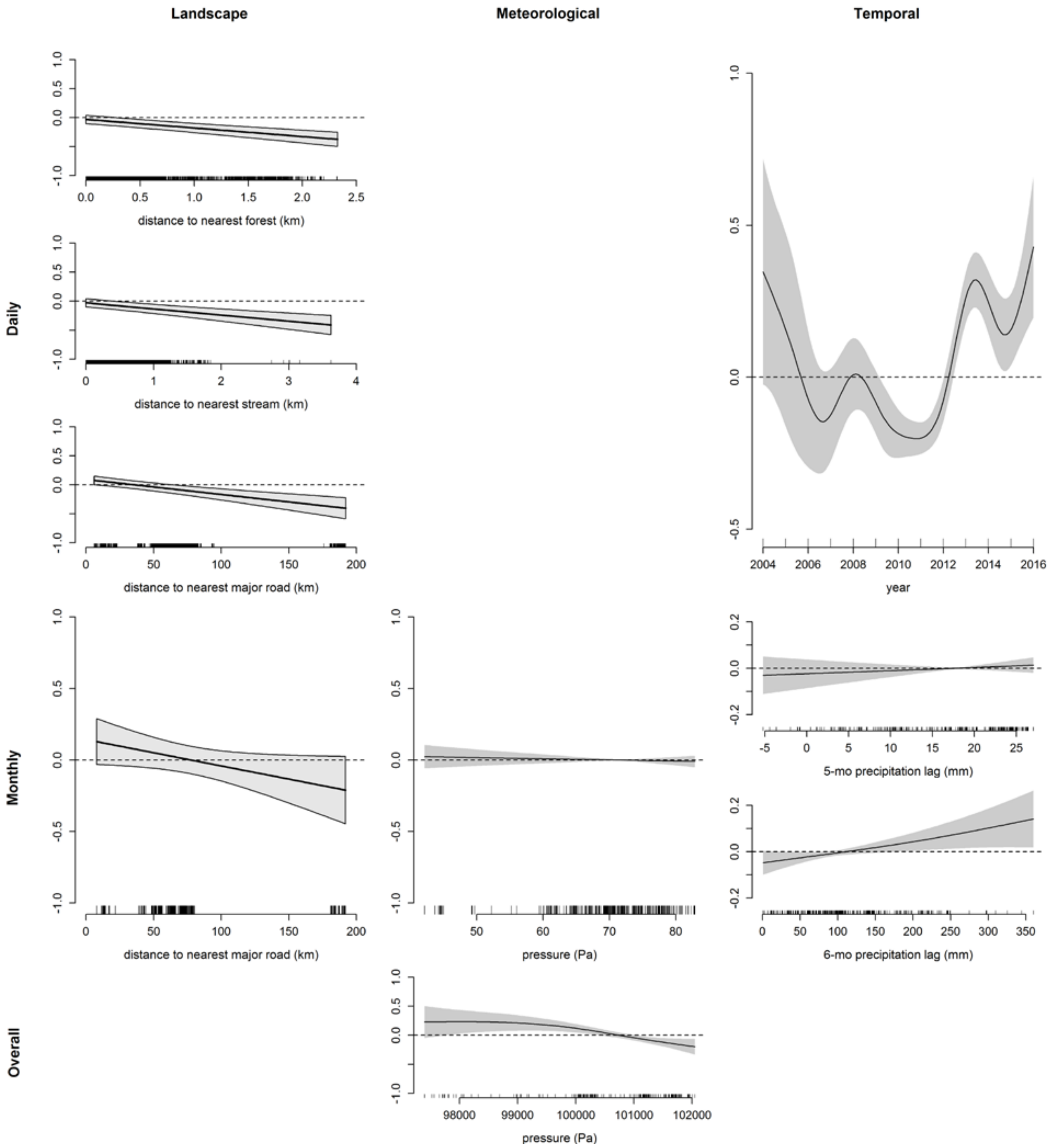

Figure S12. Relationships between significant variables not shown in the main text for the average hour distance (MHD) analyzed from wild pig location data in the southeastern U.S.A. from 2004—2016 across temporal scales. Top row depicts the daily scale, middle row shows the monthly scale, and bottom row depicts the overall scale. Columns correspond to the broad categories of predictor variables tested. The x-axes display individual variables tested while the y-axes show the deviation from the average MHD. The dashed horizontal line corresponds to the average MHD.

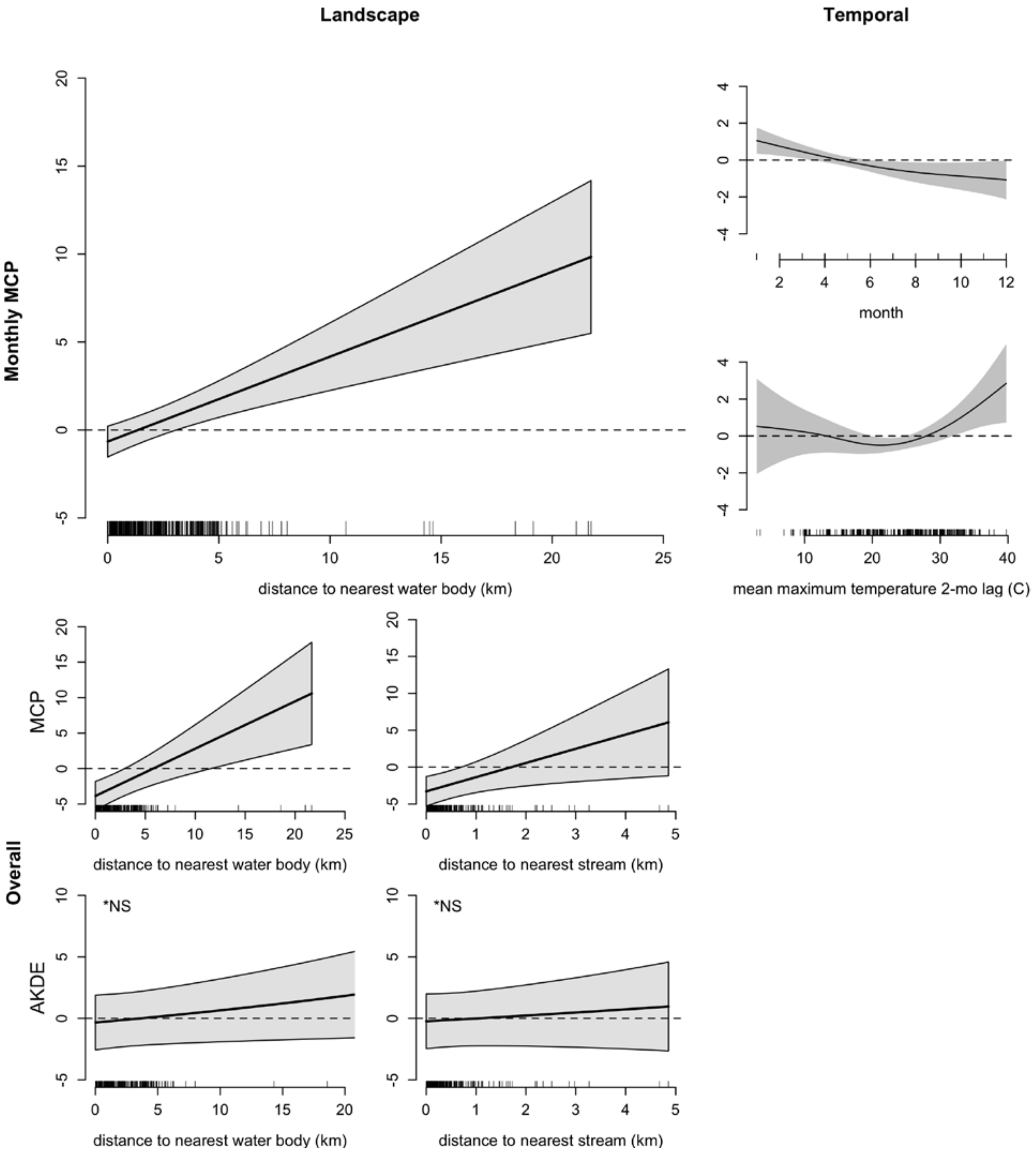

Figure S13. Relationships between significant variables not shown in the main text for the average MCP home range size analyzed from wild pig location data in the southeastern U.S.A. from 2004—2016 across temporal scales. Top row depicts the monthly scale, and bottom two rows depict the overall scale for MCP and AKDE for comparison. Plots with *NS depict non-significant relationships. Columns correspond to the broad categories of predictor variables tested. The x-axes display individual variables tested while the y-axes show the deviation from the average home range size. The dashed horizontal line corresponds to the average home range size.
